# Interrelation between Plasma Concentrations of Vitamins C and E along the Trajectory of Ageing in Consideration of Lifestyle and Body Composition: A Longitudinal Study over Two Decades

**DOI:** 10.3390/nu12102944

**Published:** 2020-09-25

**Authors:** Alexandra Jungert, Monika Neuhäuser-Berthold

**Affiliations:** 1 Institute of Nutritional Science, Justus Liebig University, Goethestr. 55, D−35390 Giessen, Germany; alexandra.jungert@ernaehrung.uni-giessen.de; 2 Interdisciplinary Research Center for Biosystems, Land Use and Nutrition (IFZ), Justus Liebig University, Heinrich-Buff-Ring 26−32, D−35392 Giessen, Germany

**Keywords:** vitamin C, ascorbic acid, vitamin E, tocopherol, body composition, longitudinal changes, elderly, physical activity, diet

## Abstract

Although the interrelation between vitamins C and E has been demonstrated on an experimental level, its impact on biomarkers in community-dwelling subjects along the trajectory of ageing has not yet been shown. The present longitudinal study investigates the determinants and interrelation of vitamins C and E plasma concentrations in 399 subjects aged ≥60 years with a median follow-up time of 12 years. Linear mixed-effects models were used to analyze the influence of age, sex, body composition, dietary intake, physical activity, smoking and supplement/drug use on plasma vitamin C, plasma α-tocopherol and α-tocopherol/total cholesterol ratio. At baseline, median plasma concentrations of vitamin C and α-tocopherol were 74 and 35 µmol/L. Absolute fat-free mass, physical activity, use of supplements, and plasma α-tocopherol were main determinants of plasma vitamin C in the course of ageing. For the α-tocopherol/total cholesterol ratio, age, use of supplements, use of lipid-modifying drugs, and plasma vitamin C were main determinants. The results reveal a stable positive interrelation between plasma concentrations of vitamins C and E along the trajectory of ageing independent of the other identified determinants. The possible regulatory mechanisms that could explain this robust positive interrelation remain to be elucidated.

## 1. Introduction

Vitamin C (ascorbic acid) and vitamin E (tocopherol) are central interacting components of the antioxidant defense system [1]. The interrelation between both vitamins is based on the ‘vitamin E recycling’: tocopherol reacts with a peroxyl radical to form a tocopheryl radical, which in turn is regenerated by vitamin C [2]. Although this interrelation has been demonstrated on an experimental level [3,4,5,6], the impact of the interrelation between both vitamins on the biomarker level in community-dwelling subjects and particularly in the course of ageing remains to be shown. 

Advancing age is frequently linked to a state of oxidative stress characterized by a disproportionate formation of reactive oxygen species without appropriate adaption of antioxidant defense mechanisms [7]. Although several studies have looked on determinants of vitamin C and/or tocopherol concentrations in blood via cross-sectional approaches [8,9,10,11,12,13,14,15], longitudinal studies investigating linear and non-linear changes of biomarkers of both vitamins and their interrelation in the course of ageing by considering changes in lifestyle, diet, serum lipids and body composition are lacking. Thus, it is still unclear whether advanced age is an independent risk factor for diminishing blood concentrations of these vitamins and whether vitamin C and/or tocopherol concentrations show linear or non-linear changes along the trajectory of ageing.

The present longitudinal study investigates determinants and the interrelation of plasma concentrations of vitamin C and α-tocopherol in community-dwelling subjects ≥60 years of age over two decades by using data from multiple follow-ups.

## 2. Materials and Methods

### 2.1. Study Design

The data of the present investigation originate from the longitudinal study on nutrition and health status in senior citizens from Giessen, Germany (GISELA study). The study design and methods were described previously [16,17,18,19]. In brief, the GISELA study was started in 1994 and completed in 2014. To participate in this study, subjects had to be at least 60 years of age, physically mobile and resident in Giessen or surrounding area. Follow-ups were conducted annually until 1998 and biennially since 1998. At each follow-up, subjects visited the Institute of Nutritional Science at the Justus Liebig University of Giessen, Germany where the investigations were carried out between July and October. The study was approved by the Ethical Committee of the Faculty of Medicine at the Justus Liebig University of Giessen, Germany (Project identification code A 30/95). Participants gave their informed consent for inclusion in the study.

### 2.2. Study Subjects

**Figure 1:**
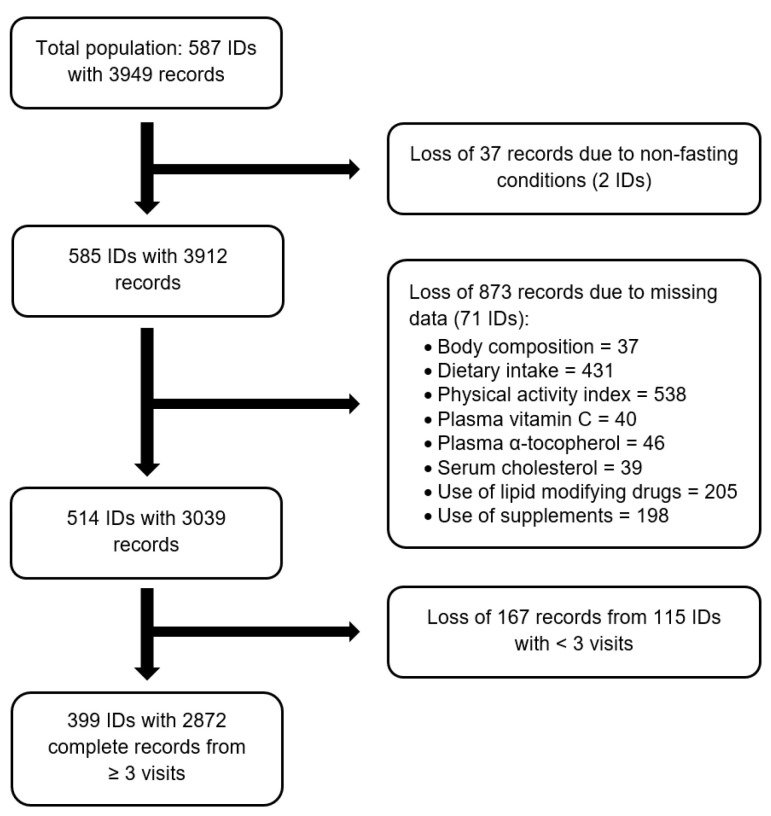
Flowchart. This flowchart illustrates the numbers and reasons for excluding subjects (indicated by IDs) and/or records. In total, 587 subjects participated in the GISELA study from 1994 to 2014. After applying the illustrated exclusion criteria, the final sample size for the present investigation was 399 subjects who had complete data records on relevant parameters on at least three visits during the study period.

From 1994 to 2006, 587 volunteers were recruited. For the present analysis, subjects who provided complete data records of relevant variables on at least three visits between 1994 and 2014 were considered. The flowchart that explains the numbers and reasons for excluding subjects and/or records is illustrated in Figure 1. Data from 399 subjects (278 women and 121 men) were analyzed. These participants visited the Institute of Nutritional Science on average 7 times (range: 3–13 visits). The follow-up period ranged between two and 20 years with a median follow-up time of 12 years.

### 2.3. Blood Biomarkers

Blood samples were collected in the morning hours after an overnight fast using serum sample tubes and heparin coated sample tubes. Samples were immediately centrifuged, and serum and plasma aliquots were stored at approximately −70 °C until analysis.

Plasma vitamin C was assessed in duplicate by photometric detection (Beckman Model 35 UV/VIS, Beckman Coulter, Inc., Krefeld, Germany until 2002; Shimadzu UV−160A, Kyoto, Japan between 2004 and 2006 and since 2008 Shimadzu UV−1800, Kyoto, Japan). The day-to-day variation was ≤4% based on pooled plasma and internal standard. Before freezing, heparin plasma samples were acidified with trichloracetic acid. Vitamin C concentrations in mg/dL were transformed in SI units (µmol/L) by multiplying with 56.7795. Vitamin C deficiency was defined as plasma concentrations < 11.4 µmol/L, and plasma concentrations < 28 µmol/L were classified as moderate risk of vitamin C deficiency [13]. An adequate vitamin C status was assumed when plasma concentrations were ≥50 µmol/L [20].

Plasma α-tocopherol was measured in duplicate by HPLC with fluorometric detection (until 2004 Gynkotek, pump model 300B, Germering, Germany and since 2006 VWR-HITACHI LaChromeElite, pump model L−2130, detector L−2400, Darmstadt, Germany). The day-to-day variation was ≤3% based on pooled plasma. Plasma α-tocopherol concentrations in mg/dL were transformed in SI units (µmol/L) by multiplying with 23.2175. Vitamin E deficiency was assumed when plasma α-tocopherol was <12 μmol/L [2,21], whereas plasma α-tocopherol ≥30 µmol/L was interpreted as adequate status [22]. As the concentrations of blood lipids and α-tocopherol show a positive relation because lipoproteins function as career for vitamin E in the circulation [21,23], plasma α-tocopherol was corrected for serum cholesterol by calculating the α-tocopherol/total cholesterol ratio (µmol/mmol).

Serum concentrations of total cholesterol were measured in duplicate by commercial enzymatic methods (CHOD-PAP) according to the manufacturer’s instructions (Roche Diagnostics GmbH, Mannheim, Germany). Cholesterol serum concentrations in mg/dL were transformed in SI units (mmol/L) by multiplying with 0.0259.

### 2.4. Anthropometric Data and Body Composition

Body mass and body height were measured at each follow-up and subsequently body mass index (BMI) (kg/m^2^) was calculated. Body composition was examined by bioelectrical impedance analysis (Akern-RJL BIA 101/S, Data Input, Frankfurt, Germany) [14].

### 2.5. Lifestyle Factors

Subjects completed at each follow-up a self-administered questionnaire on their weekly physical activity patterns and smoking behavior.

To evaluate the energy expenditures for the reported activities, we used multipliers for resting metabolic rate published by WHO [17]. The physical activity index (PAI) was calculated as the ratio of total energy expenditure for the reported activities to resting metabolic rate. The latter was measured by an open-circuit indirect calorimeter (Deltatrac^TM^ MBM−100, Hoyer, Bremen, Germany) [18].

Subjects were classified as current/ex-smokers if they reported to be current/ex-smokers at any follow-up. Subjects with partially missing data for smoking behavior were considered as non-smokers if they reported to be never-smokers at all other available measuring points.

### 2.6. Disease History and Use of Lipid-Modifying Drugs

Participants were asked via questionnaire on their diseases diagnosed by their physicians. For the present investigation, the information on the presence of gall bladder/pancreas/chronic liver/inflammatory bowel disease and dyslipidemia were combined and a dichotomous variable derived, which indicated for each participant whether the subject reported one of the above-mentioned diseases. The use of lipid-modifying drugs was coded as a dichotomous variable (no/yes).

### 2.7. Dietary Factors and Use of Supplements

Data on the use of vitamin C, vitamin E and multivitamin supplements were collected via self-administered questionnaire and coded as dummy variable into non-users and users. The subjects completed an estimated dietary record on three consecutive days [16]. The average daily dietary intakes of vitamin C, vitamin E and polyunsaturated fatty acids (PUFA) were calculated by the German Food Code and Nutrient Database version 3.02 (Max Rubner-Institute, Karlsruhe, Germany). Vitamin E intake was assessed as α-tocopherol equivalents. In addition, the ratio of mg α-tocopherol to g PUFA was calculated to account for potential differences in α-tocopherol requirements in relation to dietary intake of PUFA [24]. Dietary intakes of vitamin C and α-tocopherol equivalents of the subjects were evaluated against dietary reference values (DRVs): Population Reference Intakes (PRI) for vitamin C of 110 mg/d for males and 95 mg/d for females [20] and Adequate Intakes for vitamin E of 13 mg/d for males and 11 mg/d for females [2].

### 2.8. Statistical Analyses

The open-source software R version 3.6.3 including inter alia the packages lme4 [25], version 1.1–23, multcomp [26], version 1.4–13, and performance [27], version 0.4.5, was used for this explorative study. Results were considered statistically significant when *p* values were < 0.050.

Baseline metric data were expressed as median and 25% and 75% quartiles for the entire study population and stratified by sex. Several parameters showed deviation from normal distribution tested by Shapiro-Wilk test. Therefore, Mann-Whitney U tests were performed to investigate differences between two groups at baseline.

To investigate whether intake levels of vitamin C and α-tocopherol equivalents changed with increasing age, linear mixed-effects models were applied with linear age (centered to 72 years) and sex as fixed effects and subject and linear age as random effects. For this approach, intake levels were logarithmically transformed (natural logarithm) because of highly skewed residual distributions.

Linear mixed-effects models were used in order to examine the longitudinal influence of all relevant variables on vitamin C and α-tocopherol plasma concentrations as well as α-tocopherol/total cholesterol ratio. In these analyses, α-tocopherol plasma concentrations and α-tocopherol/total cholesterol ratios were logarithmically transformed (natural logarithm) with regard to residual distribution. Different models were created: *Model 1* included only age (centered to 72 years) as potential determinant considering fixed effects for linear and quadratic age. *Model 2* additionally included sex (female vs. male), absolute fat-free mass (FFM, centered to 42 kg for vitamin C) or fat mass (FM, centered to 27 kg for vitamin E), smoking behavior (never smoker vs. current/past smoker), PAI (centered to 1.6), use of supplements (no vs. yes), use of lipid-modifying drugs (no vs. yes), disease diagnosis (no vs. yes), plasma α-tocopherol (centered to 36 µmol/L for vitamin C) or plasma vitamin C (centered to 74 µmol/L for vitamin E) as well as linear and quadratic dietary intake of vitamin C (scaled to two-digit numbers by dividing by 10 and then centered around 10 for vitamin C) or α-tocopherol equivalents (centered to 11 mg/d for vitamin E). The metric variables were centered and scaled to reduce potential multicollinearity and to increase the numerical stability of the fitting process. *Model 3* included all variables of model 2 and in addition the following interaction terms: sex and age, sex and absolute FFM/FM, sex and nutrient intake, age and nutrient intake, smoking and nutrient intake, sex and plasma α-tocopherol/vitamin C as well as age and plasma α-tocopherol/vitamin C. Models 4 and 5 represent sensitivity analyses. *Model 4* is based on model 2, but records in which subjects reported the use of vitamin C/E containing supplements were excluded leaving 301 subjects for the vitamin C model and 289 subjects for both vitamin E models. *Model 5* is based on model 2 but only subjects who had complete data records on at least seven visits were considered, leaving 226 subjects for this analysis (median follow-up time was 15 years, range: 8–20 years). 

Based on the models 2 and 5, the following additional analyses were performed:

- For the log α-tocopherol/total cholesterol ratio: (1) The dietary variable “α-tocopherol equivalents” was replaced by the ratio of mg α-tocopherol to g PUFA (centered to 1 mg/g) to investigate whether this ratio is a better proxy for the log α-tocopherol/total cholesterol ratio than α-tocopherol equivalents. (2) Absolute FM was replaced by relative FM (centered to 39%) to investigate which body composition parameter, if any, shows a stronger association with the log α-tocopherol/total cholesterol ratio.
- For log plasma α-tocopherol: Serum cholesterol (centered to 5.6 mmol/L) was included as additional fixed effect to investigate whether the former identified predicting variables show associations with plasma α-tocopherol independent of serum cholesterol.

All linear mixed-effects models comprised subject and linear age as random effects. The conditional and marginal R^2^ were calculated as indicators for the explained variance by fixed and random effects. The presented *p* values of the linear mixed-effects models were adjusted for simultaneous inference applying methods for general linear hypotheses [26]. Diagnostics for the residuals of the fitted models indicated deviations from normal distribution as regards the QQ-normal plots for the vitamin C models and considerably heavier upper tails for the vitamin E models. Therefore, sensitivity analyses were performed, in which records with residuals > 2 were excluded.

## 3. Results

### 3.1. Baseline Characteristics 

At baseline, vitamin C and α-tocopherol plasma concentrations were in all subjects >28 µmol/L and >12 μmol/L, while 7% and 27% showed vitamin C and α-tocopherol plasma concentrations <50 µmol/L and <30 µmol/L, respectively. Dietary intakes were below PRIs for vitamin C in 34% (women) and 45% (men) of the subjects, whereas 60% (women) and 74% (men) of the subjects consumed α-tocopherol equivalents below the established Adequate Intakes. 

Baseline characteristics of the 399 investigated subjects are presented in Table 1. Women had higher plasma concentrations of vitamin C (*p* < 0.001) and α-tocopherol (*p* < 0.001) than men. No sex differences were found when the α-tocopherol/cholesterol ratio was compared (median: 6.1 vs. 5.7 µmol/L, *p* = 0.103). Subjects who reported to use vitamin C and/or multivitamin supplements had higher vitamin C plasma concentrations than non-users (77 vs. 71 µmol/L, *p* = 0.005). Likewise, subjects who reported to use vitamin E and/or multivitamin supplements had higher α-tocopherol plasma concentrations (39 vs. 33 µmol/L, *p* < 0.001) and a higher α-tocopherol/cholesterol ratio (6.6 vs. 5.7 µmol/mmol, *p* < 0.001) compared to non-users. In subjects with a diagnosis of selected diseases with possible impact on blood lipids, α-tocopherol plasma concentrations were higher (37 vs. 33 µmol/L, *p* < 0.001) than in subjects without disease diagnosis, whereas the α-tocopherol/cholesterol ratio did not significantly differ (6.1 vs. 5.9 µmol/mmol, *p* = 0.063). In subjects who used lipid-modifying drugs, α-tocopherol plasma concentrations (39 vs. 34 µmol/L, *p* < 0.001) and the α-tocopherol/cholesterol ratio (6.6 vs. 5.9 µmol/mmol, *p* < 0.001) were higher than in non-users.

### 3.2. Determinants of Plasma Concentrations of Vitamins C and E Based on a Longitudinal Approach

Before adjustments (model 1), vitamin C plasma concentrations were not affected by increasing age; neither the linear age effect (coefficient estimate [95% CI] = −0.075 [−0.194, 0.043]) nor the quadratic age effect (0.004 [−0.008, 0.015]) reached significance. For log plasma α-tocopherol concentrations, linear age showed a positive association (0.003 [0.001, 0.005]), while the quadratic age effect reached no significance (> −0.001 [> −0.001, < 0.001]). Likewise, the log α-tocopherol/cholesterol ratio was positively affected by linear age (0.009 [0.007, 0.011]), while the quadratic age effect failed to reach significance (> −0.001 [> −0.001, < 0.001]). Advancing age was associated with increasing intake levels of log α-tocopherol equivalents (0.008 [0.005, 0.011]), but showed no association with log vitamin C intake levels (0.001 [−0.002, 0.005]).

The results of the linear mixed-effects models after considering further potential influencing factors are shown in Table 2 for plasma vitamin C and in Table 3 and Table 4 for log plasma α-tocopherol and the log α-tocopherol/cholesterol ratio, respectively.

Overall, absolute FFM, PAI, use of vitamin C/multivitamin supplements and plasma α-tocopherol concentration were consistently associated with vitamin C plasma concentrations, although the association of absolute FFM with plasma vitamin C was not significant in models 4 and 5 after adjusting for multiple testing (Table 2). Neither linear nor non-linear effects of age or vitamin C intake on vitamin C plasma concentrations were found after adjusting for multiple testing. Likewise, current/past smoking and sex were no independent determinants of vitamin C plasma concentrations after adjusting for simultaneous inference. Effect modifications with respect to the considered interaction terms were not noticed (Table 2, model 3).

**Figure 2:**
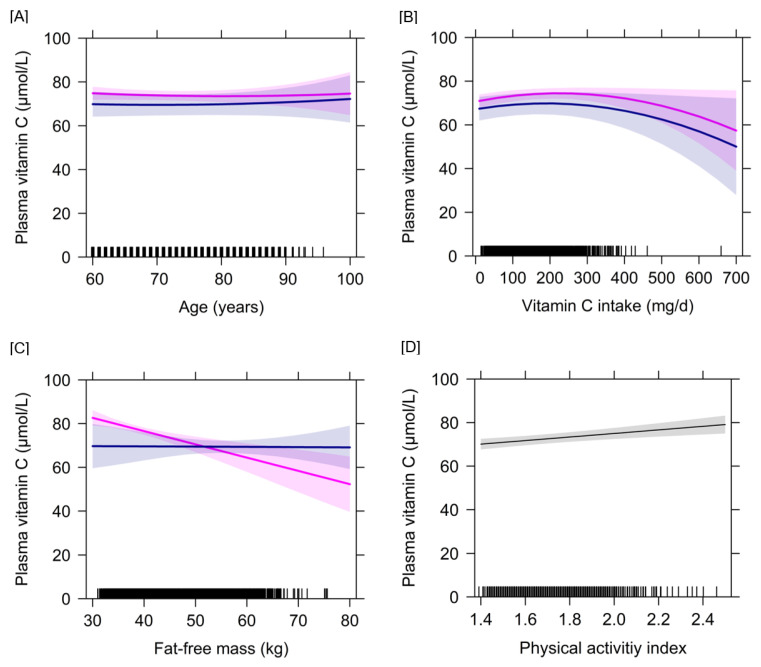
Longitudinal associations of vitamin C plasma concentrations with age, vitamin C intake, absolute fat-free mass and physical activity index separated by sex if appropriate. This figure illustrates the changes in vitamin C plasma concentrations with increasing age (**A**), vitamin C intake (**B**), fat-free mass (**C**) and physical activity index (**D**) after controlling for the cofactors included in the linear mixed-effects model 3 (*n* = 399). The thick lines represent the estimated means and the respective colored areas reflect the 95% confidence intervals. The associations are illustrated in magenta color for females (*n* = 278) and blue color for males (*n* = 121). For physical activity index, no sex-specific associations are displayed because no such interaction term was investigated.

The associations of vitamin C plasma concentrations with selected independent variables are illustrated in Figure 2. After excluding the model-wise residual outliers leading inter alia to the exclusion of the record with very high dietary vitamin C intake (>500 mg/d), the results remained largely unchanged, albeit absolute FFM became a significant negative determinant in all models (Supplementary Table S1). As indicated by the marginal and conditional R^2^, around 10–14% of the model variance was explained by fixed effects, while fixed and random effects together explained around 56–69% of the model variance.

**Figure 3:**
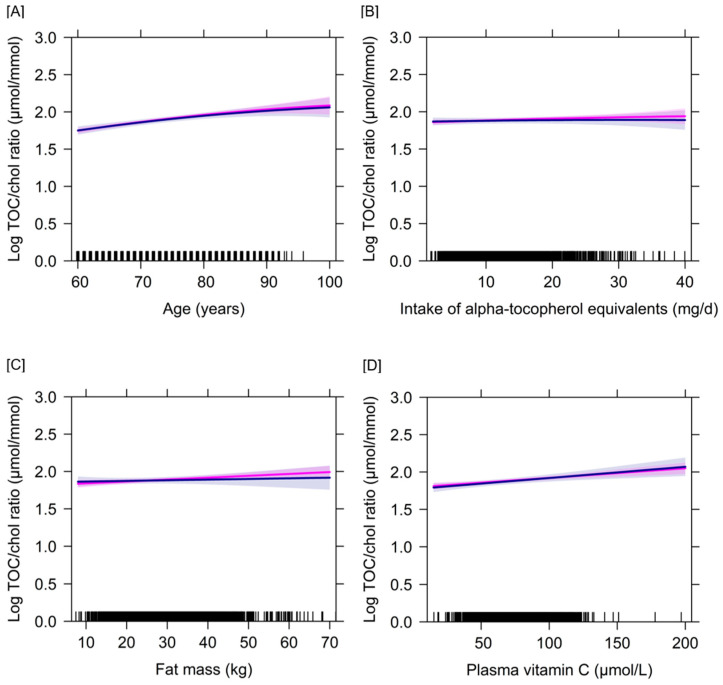
Longitudinal associations of the log α-tocopherol/total cholesterol ratio with age, intake of α-tocopherol equivalents, absolute fat mass and vitamin C plasma concentrations separated by sex. This figure illustrates the changes in the log α-tocopherol/total cholesterol ratio with increasing age (**A**), intake of α-tocopherol equivalents (**B**), fat mass (**C**) and vitamin C plasma concentrations (**D**) after controlling for the cofactors included in the linear mixed-effects model 3 (*n* = 399). The thick lines represent the estimated means and the respective colored areas reflect the 95% confidence intervals. The associations are illustrated in magenta color for females (*n* = 278) and blue color for males (*n* = 121). Abbreviation: TOC/chol ratio = α-tocopherol/total cholesterol ratio.

For log plasma α-tocopherol concentrations, linear age, male sex, use of vitamin E/multivitamin supplements, use of lipid-modifying drugs, plasma vitamin C and disease diagnosis were identified as significant determinants (Table 3). The significant effects of sex and lipid-modifying drugs vanished in three of the four models and the effect of disease was not found in models 4 and 5 after including serum cholesterol as additional fixed effect, while the other identified determinants remained significant in addition to serum cholesterol (Supplementary Table S2). Concerning the determinants of the log α-tocopherol/cholesterol ratio, linear age, use of vitamin E/multivitamin supplements, use of lipid-modifying drugs and plasma vitamin C were robust determinants after adjusting for simultaneous inference (Table 4). Sex, absolute FM, intake of α-tocopherol equivalents, smoking behavior, PAI and disease history were no independent determinants of the log α-tocopherol/cholesterol ratio. Effect modifications with respect to the considered interaction terms were not noticed. The associations of the log α-tocopherol/cholesterol ratio with selected independent variables are illustrated in Figure 3. The replacement of the dietary variable “α-tocopherol equivalents” with the α-tocopherol/PUFA ratio yielded comparable results indicating no significant effect of the α-tocopherol/PUFA ratio on the log α-tocopherol/cholesterol ratio (data not shown). Likewise, when absolute FM was replaced by relative FM, relative FM was no significant determinant and no substantial changes in the results were found concerning the previously identified independent determinants of the log α-tocopherol/cholesterol ratio (data not shown). The same determinants for log plasma α-tocopherol concentrations and the log α-tocopherol/cholesterol ratio were identified after excluding model-wise residual outliers (Supplementary Tables S3 and S4). As indicated by the marginal R^2^, the fixed effects explained around 11–34% of the model variance, while fixed and random effects together explained around 62–80% of the model variance. More variance was explained for log plasma α-tocopherol than for the log α-tocopherol/cholesterol ratio.

## 4. Discussion

The unique feature of the present longitudinal study is the investigation of the trajectories and interrelation of vitamin C and α-tocopherol plasma concentrations as a function of lifestyle factors in community-dwelling elderly subjects during their course of advanced ageing by covering two decades of lifespan.

### 4.1. Determinants of Vitamin C Biomarker Status in the Course of Ageing

The present study shows that supplemental vitamin C intake, plasma α-tocopherol, body composition and physical activity are the main determinants for the trajectory of plasma vitamin C along the course of ageing.

The mean dietary vitamin C intake of 117 mg/d of the GISELA subjects at baseline is comparable or rather at the upper end of intakes observed in European populations and is above DRVs [20]. Only 7% of the subjects had plasma vitamin C concentrations below 50 µmol/L, the level assumed to reflect saturation of body pools and an adequate vitamin C status [20]. The observation that the vitamin C plasma concentration progressively reaches a plateau at plasma values above 50 µmol/L [20] may explain the lacking association between dietary intake and plasma vitamin C in our cohort. Figure 2 reveals a non-linear association between vitamin C intake and plasma vitamin C with parallel trajectories for women and men, but the wide 95% CIs illustrate the level of uncertainty in the estimates at high vitamin C intakes.

In contrast to dietary vitamin C intake, the use of supplements proves as determinant of vitamin C status irrespective of the overall adequate dietary supply with vitamin C. This finding is in line with observations that vitamin C intakes above 200 mg/day may progressively elevate the plasma concentration to a plateau at 70–80 µmol/L or even above [20]. In a previous cross-sectional analysis on the supplement dosages used by the GISELA subjects, supplements provided additional vitamin C in amounts equal to or above DRVs [28]. 

Although vitamin C intake does not differ between female and male GISELA subjects, plasma vitamin C concentrations are consistently higher in women than in men throughout the course of ageing. The graphical illustration of the longitudinal association between plasma vitamin C and age shows remarkable stable and parallel trajectories of plasma vitamin C for women and men, which approximate each other in the very advanced age (Figure 2). It is a common observation that men have a lower vitamin C status than women. Apart from differences in lifestyle factors, this finding has been attributed to differences in body weight and body composition and resultant changes in the dilution volume [14,29]. Of note, the present study does not only indicate a negative association of absolute FFM with plasma vitamin C on the long-term but also a notable (and, before adjusting for simultaneous inference, significant) interaction between sex and absolute FFM on the trajectory of plasma vitamin C. While the trajectory of plasma vitamin C in males seems unaffected by increasing FFM, a higher absolute FFM leads to a strong decrease in plasma vitamin C in females. Considering the 95% CI of the females, one can speculate that that the effect in females is predominantly caused by their generally lower absolute FFM compared to males.

Although vitamin C is expected to affect ageing processes and related diseases due to its well-known widespread antioxidant functions in the body [30], there is little information on the nature of the trajectory of vitamin C status and its influencing factors during advanced ageing in humans. In a cross-sectional study in hospitalized patients, plasma ascorbic acid was significantly lower in persons > 75 years of age compared with younger subjects [31]. In contrast, no cross-sectional association between plasma vitamin C and age was found in a large European cohort [32]. Based on our longitudinal study, there is no age-related decline in vitamin C biomarker status in community-dwelling elderly with a median vitamin C intake around DRVs.

PAI presents as a robust positive determinant of plasma vitamin C along the course of ageing. Physical activities of the GISELA subjects mainly comprise housework, gardening and recreational activities of moderate intensity rather than exhaustive physical exercises [17]. While exhaustive exercise can cause oxidative stress, moderate to strenuous exercise has been shown to significantly elevate endogenous antioxidant defenses [33,34,35] and thereby might save circulating exogenous antioxidants such as vitamin C. In cross-sectional studies, a low level of physical activity was identified among the risk factors for vitamin C deficiency [36] and plasma vitamin C was positively associated with handgrip strength and physical performance [37]. Some studies found beneficial effects of supplemental vitamin C on physical performance in subjects with initially low plasma vitamin C [38,39]. However, such effects are generally not seen in healthy persons with an adequate vitamin C status [38,39,40]. The results of our study do not allow conclusions on whether PAI affects plasma vitamin C or vice versa. It may be speculated that the association reflects an overall good physical condition associated with higher vitamin C status and less oxidative stress.

Although we observed a negative association of plasma vitamin C with smoking in some of the models, the association failed to reach significance after applying simultaneous inference procedures. Smoking is considered a source of oxidative stress and has been associated with lower plasma concentrations of vitamin C and carotenoids, albeit current smokers seem to exhibit a stronger decrease in vitamin C than past smokers [41]. The reason for the lacking association in our cohort may be that current and past smokers were assigned to one category and only a few subjects reported to be current smokers.

### 4.2. Determinants of Vitamin E Biomarker Status in the Course of Ageing

The present study shows that age, use of supplements, plasma vitamin C and lipid-modifying drugs are the main determinants of the trajectories of plasma α-tocopherol and α-tocopherol/cholesterol ratio along the course of ageing, while diseases related to dyslipidemias were associated with plasma α-tocopherol but not with the α-tocopherol/cholesterol ratio.

With regard to vitamin E intakes and plasma concentrations of the GISELA subjects at baseline, the levels are comparable to those observed in Germany [42] and other European populations [2]. Although vitamin E intakes are slightly below the values set for an adequate intake, median plasma concentrations are considerably above the deficiency cut-off value of 12 µmol/L in both sexes. In our study, dietary α-tocopherol intake is not a robust determinant of the trajectory of plasma α-tocopherol along the course of ageing after adjusting for simultaneous inference, irrespective of the consideration of PUFA intake. This finding is in agreement with a review of the literature that came to the conclusion that the plasma/serum α-tocopherol concentration is not a sensitive marker of dietary α-tocopherol intake [2].

Contrary to dietary intake of α-tocopherol, the use of supplements is positively associated with plasma α-tocopherol in our study, which may be the consequence of a total intake considerably above DRVs. As described elsewhere, median (5th–95th percentile) intake of supplemental vitamin E of the GISELA subjects was 134 (3–404) mg/d for females and 36 (10–386) mg/d for males [28]. In a study in seven healthy men who received a diet low in α-tocopherol and supplements containing up to 800 mg α-tocopherol per day, plasma α-tocopherol markedly increased at dosages up to 150 mg/d and leveled off at dosages > 350 mg/d [43]. 

In contrast to vitamin C, linear age is an independent positive determinant of both plasma α-tocopherol and α-tocopherol/cholesterol ratio along the course of ageing, which is in line with results from several previous studies reporting an increase of plasma α-tocopherol with increasing age [44,45,46,47,48,49]. However, none of these studies used multiple follow-ups and allowed to investigate non-linear trajectories of plasma α-tocopherol and/or α-tocopherol/cholesterol ratio in the course of ageing. The age-related changes in vitamin E biomarker status have been linked to an age-related increase in cholesterol concentrations, which have been shown to be a major determinant of the vitamin E status [50,51]. However, in the present study, the positive age effect persists even after adjusting for serum cholesterol. This finding might be a result of a selection bias and/or a more health conscious behavior in advanced age as indicated in a previous study [52]. Although we found an age-related increase in vitamin E intake, vitamin E intake itself was not identified as a main determinant of plasma α-tocopherol and there was no substantial interaction between age and vitamin E intake. Whether the positive age effect can be attributed to age-related changes in vitamin E metabolism or to diseases and medications needs further exploration. Interestingly, α-tocopherol concentrations are significantly lower in men than in women but not when investigated in relation to circulating cholesterol concentrations. Thus, the initially observed sex differences could be due to the somewhat lower cholesterol concentrations in the male subjects of our study. The median serum cholesterol concentration in our subjects is within the sex-specific reference ranges reported for the age group 66–100 years, which also indicate lower values for men compared to women [53]. Furthermore, in our study, a lower percentage of men compared to women takes lipid-modifying drugs and suffers from diseases with possible impact on blood lipids. The use of lipid-modifying drugs is associated with lower plasma α-tocopherol but a higher α-tocopherol/cholesterol ratio. However, the effect of lipid-modifying drugs on plasma α-tocopherol in most cases vanishes after adjusting for serum cholesterol. Assuming that the use of such drugs lowers serum cholesterol, the decrease in serum cholesterol results in a decrease in circulating α-tocopherol, although the decrease is more pronounced in serum cholesterol than in plasma α-tocopherol what explains the positive association of lipid-modifying drugs with the α-tocopherol/cholesterol ratio. This is corroborated by results from a meta-analysis, which indicated a significant reduction in plasma vitamin E following statin treatment, whereas cholesterol adjusted vitamin E concentrations, defined as vitamin E/cholesterol ratio, were found to be improved by statin therapy [54]. The observed higher plasma α-tocopherol in subjects with at least one of the reported diseases may arise from the higher cholesterol concentrations in those subjects, as the α-tocopherol/cholesterol ratio is unaffected. Another reason for this finding might be that the regulation of plasma α-tocopherol differs in the presence of hyperlipidemia, such that the uptake of α-tocopherol into peripheral tissues may be reduced [55].

### 4.3. Interrelation between Vitamins C and E

Our study demonstrates a robust positive interrelation between vitamins C and E status in community-dwelling elderly subjects during the course of advanced ageing, even in subjects not taking supplements. It is well known that α-tocopherol and ascorbic acid act synergistically in counteracting free radicals where α-tocopherol is able to quench free radicals in a hydrophobic environment and the resulting tocopherol radical is then regenerated by ascorbic acid [56]. Most of this evidence is based on animal and in-vitro studies [3,4,6] and studies investigating this relationship in vivo are scarce. One study providing pharmacological doses of either vitamin C or E to a group of healthy middle-aged adults for six weeks showed an increase in fasting plasma ascorbic acid after supplementation with α-tocopherol and an increased vitamin E status after ascorbic acid supplementation [5]. In another intervention study, pharmacological doses of vitamin C for two weeks attenuated fractional disappearance rates of plasma α- and γ-tocopherol in smokers, indicating that plasma vitamin C reduces α- and γ-tocopheroxyl radicals resulting from oxidative stress due to smoking [57]. We are not aware of any non-interventional study demonstrating the interrelation between these two vitamins in community-dwelling older adults in the course of ageing after controlling for relevant cofactors. Importantly, the interrelation is not modified by increasing age or sex in the present study. Although one may assume, that the cause of the positive impact of plasma vitamin C on α-tocopherol may depend on its regenerating action on α-tocopherol, the mechanisms behind this positive interrelation remain to be further elucidated, in particular, regarding the observed positive impact of vitamin E supplements on plasma vitamin C [5]. This is important as vitamin E protects blood lipids from oxidation and thereby plays a role in the development of atherosclerotic and cardiovascular diseases [58]. However, as demonstrated in in-vitro studies, its protecting effects depend on the reducing action of vitamin C, which prevents α-tocopherol becoming a pro-oxidant [59]. In consideration of the observed strong mutual association between plasma vitamin C and α-tocopherol, one may speculate that mechanisms are involved in balancing plasma concentrations of vitamin C and α-tocopherol to a certain degree that prevents oxidation of α-tocopherol.

### 4.4. Study Limitations and Strengths

The interpretation of the present findings should consider some limitations. We measured only plasma α-tocopherol as this has been identified as the major vitamin E form in plasma in European populations including Germany [2]. We did not include measurements of other components of oxidant or antioxidant status that may affect or be related to either plasma vitamin C or E. Data on disease history, medication, physical activity and dietary intake relied on self-administered questionnaires and estimated dietary records. Disease diagnoses were summarized and not differentiated based on severity or incidence of the disease. 

For the strengths, we want to point out that this study includes multiple follow-ups over two decades on both biomarkers as well as body composition and lifestyle factors including physical activity, smoking and dietary intake. Plasma concentrations of vitamins C and E as well as body composition and lifestyle factors were assessed by using the same methods over the study period. The calculation of PAI values was based on measured resting energy expenditure. For the dietary assessment, we used a tool especially developed for the GISELA study and validated for protein and energy intake. Non-linear effects as well as effect modifications were investigated, sensitivity analyses were performed and simultaneous inference procedures were applied.

## 5. Conclusions

The results of this longitudinal study demonstrate that plasma concentrations of vitamins C and E can be maintained within the accepted reference ranges with regular diets and an active lifestyle along the trajectory of advanced ageing. There is a stable positive interrelation between plasma concentrations of vitamins C and E in community-dwelling older adults under every day conditions along the trajectory of ageing. Although the use of supplements, physical activity, body composition, serum cholesterol, diseases and drugs may modify plasma concentrations of either vitamin C or E, our data indicate that the interrelation between the plasma concentrations of both vitamins is largely independent of these factors. In consideration of the importance of vitamins C and E in combatting oxidative stress and age-related diseases it may be worthwhile to explore possible regulatory mechanisms that could explain this robust positive interrelation.

## Figures and Tables

**Table 1:** Descriptive characteristics of the GISELA subjects at baseline ^a^.

Parameter	Total (*n* = 399)		Women (*n* = 278)		Men (*n* = 121)	
	Median	Q25, Q75	Median	Q25, Q75	Median	Q25, Q75
Age (years)	66	62, 70	67	62, 71	66	63, 70
Body mass index (kg/m^2^)	26	24, 29	26	24, 29	26	24, 28
Fat-free mass (kg)	42	38, 52	39	38, 42	54	52, 58
Fat mass (kg)	27	22, 33	28	23, 35	23	19, 29
Fat mass (%)	39	33, 44	42	38, 46	30	27, 33
Plasma vitamin C (µmol/L)	74	64, 85	77	67, 88	68	60, 76
Plasma α-tocopherol (µmol/L)	35	29, 42	36	30, 42	32	27, 39
Serum cholesterol (mmol/L)	5.8	5.2, 6.4	5.9	5.3, 6.5	5.6	4.7, 6.2
Energy intake (MJ/d)	8.5	7.1, 10	8.1	6.8, 9.6	9.9	8.0, 12
Vitamin C intake (mg/d)	117	84, 152	117	84, 156	117	83, 138
Vitamin E intake (mg/d) ^b^	9.9	7.5, 14	9.8	7.4, 14	10	8.0, 13
PUFA intake (g/d)	10	7.7, 13	9.6	7.1, 13	11	8.4, 14
Physical activity index	1.7	1.6, 1.8	1.7	1.6, 1.8	1.7	1.6, 1.8
	***n***	**%**	***n***	**%**	***n***	**%**
Female sex	278	70				
Vitamin C supplement users	112	28	81	29	31	26
Vitamin E supplement users	107	27	81	29	26	22
Multivitamin supplement users	104	26	79	28	25	21
Current/past smokers	178	45	86	31	92	76
Users of lipid-modifying drugs	63	16	48	17	15	12
Disease diagnosis ^c^	236	59	180	65	56	46

PUFA, polyunsaturated fatty acids. ^a^ Data are presented as median and interquartile range indicated by 25% (Q25) and 75% (Q75) quartiles for continuous variables and absolute and relative frequencies for categorical variables, respectively; ^b^ Vitamin E intake is expressed in form of α-tocopherol equivalents; ^c^ This variable combined the information on reported diagnoses of gall bladder/pancreas/chronic liver/inflammatory bowel disease and dyslipidemia.

**Table 2:** Results of linear mixed-effects models for determinants of plasma vitamin C concentrations ^a,b^.

	Model 2 (*n* = 399)		Model 3 (*n* = 399)		Model 4 (*n* = 301)		Model 5 (*n* = 226)	
	CE	[95% CI]	CE	[95% CI]	CE	[95% CI]	CE	[95% CI]
Intercept	7.5 × 10^+1^ ***	[7.3 × 10^+1^, 7.8 × 10^+1^]	7.5 × 10^+1^ ***	[7.2 × 10^+1^, 7.7 × 10^+1^]	7.5 × 10^+1^ ***	[7.3 × 10^+1^, 7.8 × 10^+1^]	7.5 × 10^+1^ ***	[7.2 × 10^+1^, 7.9 × 10^+1^]
Age (years)	−3.2 × 10^−2^	[−1.5 × 10^−1^, 8.9 × 10^−2^]	−7.6 × 10^−2^	[−2.3 × 10^−1^, 7.5 × 10^−2^]	−6.8 × 10^−2^	[−2.1 × 10^−1^, 7.3 × 10^−2^]	−1.4 × 10^−2^	[−1.5 × 10^−1^, 1.2 × 10^−1^]
Age^2^ (years)	3.4 × 10^−3^	[−8.1 × 10^−3^, 1.5 × 10^−2^]	3.1 × 10^−3^	[−8.5 × 10^−3^, 1.5 × 10^−2^]	1.2 × 10^−3^	[−1.3 × 10^−2^, 1.5 × 10^−2^]	4.4 × 10^−3^	[−8.2 × 10^−3^, 1.7 × 10^−2^]
Male sex	−1.6 × 10^+0^	[−6.4 × 10^+0^, 3.2 × 10^+0^]	−5.8 × 10^+0^	[−1.2 × 10^+1^, 2.6 × 10^−1^]	−1.8 × 10^+0^	[−7.1 × 10^+0^, 3.5 × 10^+0^]	−2.4 × 10^+0^	[−8.1 × 10^+0^, 3.5 × 10^+0^]
FFM (kg)	−3.7 × 10^−1^ *	[−6.2 × 10^−1^, −1.3 × 10^−1^]	−6.1 × 10^−1^ **	[−9.2 × 10^−1^, −3.0 × 10^−1^]	−3.7 × 10^−1 ‡^	[−6.5 × 10^−1^, −9.5 × 10^−2^]	−3.3 × 10^−1^	[−6.2 × 10^−1^, −3.8 × 10^−2^]
Current/past smoking	−3.0 × 10^+0^	[−5.8 × 10^+0^, −2.0 × 10^−1^]	−3.2 × 10^+0^	[−6.0 × 10^+0^, −3.6 × 10^−1^]	−2.0 × 10^+0^	[−5.0 × 10^+0^, 1.0 × 10^+0^]	−2.3 × 10^+0^	[−5.7 × 10^+0^, 1.1 × 10^+0^]
PAI	8.3 × 10^+0^ **	[4.1 × 10^+0^, 1.3 × 10^+1^]	8.2 × 10^+0^ **	[3.9 × 10^+0^, 1.2 × 10^+1^]	9.8 × 10^+0^ **	[4.6 × 10^+0^, 1.5 × 10^+1^]	7.5 × 10^+0^ *	[2.4 × 10^+0^, 1.2 × 10^+1^]
Vitamin C intake (mg/d)	1.8 × 10^−1^	[4.0 × 10^−2^, 3.2 × 10^−1^]	1.6 × 10^−1^	[−1.2 × 10^−2^, 3.3 × 10^−1^]	1.5 × 10^−1^	[−2.6 × 10^−2^, 3.2 × 10^−1^]	1.7 × 10^−1^	[−4.1 × 10^−3^, 3.5 × 10^−1^]
Vitamin C intake^2^ (mg/d)	−7.8 × 10^−3^	[−1.4 × 10^−2^, −1.3 × 10^−3^]	−7.6 × 10^−3^	[−1.4 × 10^−2^, −1.0 × 10^−3^]	−6.7 × 10^−3^	[−1.7 × 10^−2^, 3.0 × 10^−3^]	−5.1 × 10^−3^	[−1.5 × 10^−2^, 5.0 × 10^−3^]
Use of supplements ^c^	3.9 × 10^+0^ ***	[2.7 × 10^+0^, 5.2 × 10^+0^]	3.9 × 10^+0^ ***	[2.7 × 10^+0^, 5.2 × 10^+0^]			4.3 × 10^+0^ ***	[2.9 × 10^+0^, 5.8 × 10^+0^]
Use of lipid-modifying drugs	9.8 × 10^−2^	[−1.6 × 10^+0^, 1.8 × 10^+0^]	1.9 × 10^−1^	[−1.5 × 10^+0^, 1.9 × 10^+0^]	−7.3 × 10^−1^	[−2.8 × 10^+0^, 1.3 × 10^+0^]	−2.7 × 10^−1^	[−2.2 × 10^+0^, 1.7 × 10^+0^]
Plasma TOC (µmol/L)	1.5 × 10^−1^ ***	[9.1 × 10^−2^, 2.0 × 10^−1^]	1.3 × 10^−1^ **	[6.9 × 10^−2^, 2.0 × 10^−1^]	2.0 × 10^−1^ ***	[1.2 × 10^−1^, 2.7 × 10^−1^]	2.0 × 10^−1^ ***	[1.3 × 10^−1^, 2.7 × 10^−1^]
Disease diagnosis ^d^	−1.2 × 10^+0^	[−3.9 × 10^+0^, 1.4 × 10^+0^]	−1.0 × 10^+0^	[−3.7 × 10^+0^, 1.7 × 10^+0^]	−2.3 × 10^+0^	[−5.2 × 10^+0^, 5.5 × 10^−1^]	−1.4 × 10^+0^	[−4.7 × 10^+0^, 2.0 × 10^+0^]
I (male sex:age)			6.3 × 10^−2^	[−1.9 × 10^−1^, 3.2 × 10^−1^]				
I (male sex:FFM)			6.0 × 10^−1^	[1.0 × 10^−1^, 1.1 × 10^+0^]				
I (male sex:VC intake)			−5.6 × 10^−2^	[−2.9 × 10^−1^, 1.8 × 10^−1^]				
I (age:VC intake)			5.0 × 10^−3^	[−8.3 × 10^−3^, 1.8 × 10^−2^]				
I (smoking:VC intake)			6.9 × 10^−2^	[−1.4 × 10^−1^, 2.8 × 10^−1^]				
I (male sex:plasma TOC)			6.4 × 10^−2^	[−6.7 × 10^−2^, 1.9 × 10^−1^]				
I (age:plasma TOC)			4.6 × 10^−3^	[−3.2 × 10^−3^, 1.2 × 10^−2^]				
R^2^, marginal/conditional	0.107/0.571		0.112/0.574		0.104/0.561		0.121/0.573	

CE, coefficient estimate; 95% CI, 95% confidence interval; FFM, fat-free mass; PAI, physical activity index; TOC, α-tocopherol; VC, vitamin C; I (a:b) denotes the interaction effect for a and b. ^a^ Linear mixed-effects models including plasma vitamin C concentrations as dependent variable, random effects of age and subject and centered metric independent variables. Model 2 considered as fixed effects: linear and quadratic age, sex, FFM, smoking behavior, PAI, linear and quadratic dietary vitamin C intake, use of supplements, use of lipid-modifying drugs, plasma α-tocopherol and disease diagnosis. Model 3 is based on model 2 and included interaction effects as additional fixed effects. Model 4 is based on model 2 but excluded records, in which the use of vitamin C and/or multivitamin supplements was reported. Model 5 is based on model 2 but only subjects with complete data records on at least seven visits were considered. Data are presented as coefficient estimates and 95% confidence intervals; ^b^ *p* values after adjusting for multiple testing: ^‡^ *p* < 0.10; * *p* < 0.05; ** *p* < 0.01; *** *p* < 0.001; ^c^ This variable comprised the use of vitamin C and/or multivitamin supplements; ^d^ This variable combined the information on reported diagnoses of gall bladder/pancreas/chronic liver/inflammatory bowel disease and dyslipidemia.

**Table 3:** Results of linear mixed-effects models for determinants of log plasma α-tocopherol concentrations ^a,b^.

	Model 2 (*n* = 399)		Model 3 (*n* = 399)		Model 4 (*n* = 289)		Model 5 (*n* = 226)	
	CE	[95% CI]	CE	[95% CI]	CE	[95% CI]	CE	[95% CI]
Intercept	3.5 × 10^+0^ ***	[3.5 × 10^+0^, 3.6 × 10^+0^]	3.5 × 10^+0^ ***	[3.5 × 10^+0^, 3.6 × 10^+0^]	3.5 × 10^+0^ ***	[3.5 × 10^+0^, 3.5 × 10^+0^]	3.5 × 10^+0^ ***	[3.5 × 10^+0^, 3.6 × 10^+0^]
Age (years)	5.1 × 10^−3^ ***	[3.3 × 10^−3^, 7.0 × 10^−3^]	6.0 × 10^−3^ ***	[3.8 × 10^−3^, 8.1 × 10^−3^]	5.5 × 10^−3^ ***	[3.6 × 10^−3^, 7.3 × 10^−3^]	6.6 × 10^−3^ ***	[4.6 × 10^−3^, 8.6 × 10^−3^]
Age^2^ (years)	−1.8 × 10^−4^	[−3.5 × 10^−4^, −1.7 × 10^−5^]	−1.6 × 10^−4^	[−3.3 × 10^−4^, 1.2 × 10^−5^]	−2.0 × 10^−4^	[−3.7 × 10^−4^, −2.0 × 10^−5^]	−1.9 × 10^−4^	[−3.7 × 10^−4^, −1.5 × 10^−5^]
Male sex	−1.1 × 10^−1^ ***	[−1.6 × 10^−1^, −5.6 × 10^−2^]	−1.1 × 10^−1^ **	[−1.6 × 10^−1^, −5.1 × 10^−2^]	−1.1 × 10^−1^ **	[−1.6 × 10^−1^, −5.2 × 10^−2^]	−1.5 × 10^−1^ ***	[−2.1 × 10^−1^, −7.9 × 10^−2^]
FM (kg)	6.1 × 10^−4^	[−1.4 × 10^−3^, 2.6 × 10^−3^]	7.4 × 10^−4^	[−1.6 × 10^−3^, 3.1 × 10^−3^]	1.0 × 10^−5^	[−2.1 × 10^−3^, 2.1 × 10^−3^]	−2.8 × 10^−4^	[−2.8 × 10^−3^, 2.2 × 10^−3^]
Current/past smoking	4.2 × 10^−2^	[−6.2 × 10^−3^, 9.0 × 10^−2^]	4.3 × 10^−2^	[−4.6 × 10^−3^, 9.1 × 10^−2^]	3.4 × 10^−2^	[−1.4 × 10^−2^, 8.3 × 10^−2^]	4.8 × 10^−2^	[−1.2 × 10^−2^, 1.1 × 10^−1^]
PAI	2.6 × 10^−2^	[−3.4 × 10^−2^, 8.5 × 10^−2^]	2.7 × 10^−2^	[−3.3 × 10^−2^, 8.7 × 10^−2^]	1.9 × 10^−2^	[−4.5 × 10^−2^, 8.3 × 10^−2^]	9.1 × 10^−2^	[2.3 × 10^−2^, 1.6 × 10^−1^]
TOC intake (mg/d)	2.3 × 10^−3^	[2.1 × 10^−4^, 4.4 × 10^−3^]	3.5 × 10^−3^	[9.9 × 10^−4^, 6.1 × 10^−3^]	5.3 × 10^−4^	[−1.7 × 10^−3^, 2.8 × 10^−3^]	2.8 × 10^−3^	[4.4 × 10^−4^, 5.2 × 10^−3^]
TOC intake^2^ (mg/d)	−9.6 × 10^−5^	[−2.5 × 10^−4^, 5.5 × 10^−5^]	−6.7 × 10^−5^	[−2.2 × 10^−4^, 9.0 × 10^−5^]	−2.4 × 10^−5^	[−1.8 × 10^−4^, 1.3 × 10^−4^]	−1.7 × 10^−4^	[−3.5 × 10^−4^, 7.8 × 10^−6^]
Use of supplements ^c^	1.2 × 10^−1^ ***	[1.0 × 10^−1^, 1.4 × 10^−1^]	1.2 × 10^−1^ ***	[1.0 × 10^−1^, 1.4 × 10^−1^]			1.1 × 10^−1^ ***	[8.7 × 10^−2^, 1.3 × 10^−1^]
Use of lipid-modifying drugs	−5.4 × 10^−2^ ***	[−7.8 × 10^−2^, −3.0 × 10^−2^]	−5.4 × 10^−2^ ***	[−7.8 × 10^−2^, −2.9 × 10^−2^]	−3.1 × 10^−2^	[−5.7 × 10^−2^, −3.5 × 10^−3^]	−5.6 × 10^−2^ ***	[−8.3 × 10^−2^, −2.9 × 10^−2^]
Plasma vitamin C (µmol/L)	1.6 × 10^−3^ ***	[1.1 × 10^−3^, 2.1 × 10^−3^]	1.5 × 10^−3^ ***	[9.0 × 10^−4^, 2.1 × 10^−3^]	1.7 × 10^−3^ ***	[1.1 × 10^−3^, 2.3 × 10^−3^]	1.8 × 10^−3^ ***	[1.2 × 10^−3^, 2.4 × 10^−3^]
Disease diagnosis ^d^	1.1 × 10^−1^ ***	[6.9 × 10^−2^, 1.6 × 10^−1^]	1.1 × 10^−1^ ***	[6.8 × 10^−2^, 1.6 × 10^−1^]	7.5 × 10^−2^ *	[3.0 × 10^−2^, 1.2 × 10^−1^]	9.2 × 10^−2^ *	[3.4 × 10^−2^, 1.5 × 10^−1^]
I (male sex:age)			−2.4 × 10^−3^	[−6.1 × 10^−3^, 1.4 × 10^−3^]				
I (male sex:FM)			−3.5 × 10^−4^	[−4.9 × 10^−3^, 4.2 × 10^−3^]				
I (male sex:TOC intake)			−9.1 × 10^−4^	[−4.8 × 10^−3^, 3.0 × 10^−3^]				
I (age:TOC intake)			−1.1 × 10^−4^	[−3.3 × 10^−4^, 1.0 × 10^−4^]				
I (smoking:TOC intake)			−2.2 × 10^−3^	[−5.9 × 10^−3^, 1.4 × 10^−3^]				
I (male sex:plasma vitamin C)			2.8 × 10^−4^	[−8.1 × 10^−4^, 1.4 × 10^−3^]				
I (age:plasma vitamin C)			−3.0 × 10^−5^	[−9.8 × 10^−5^, 3.8 × 10^−5^]				
R^2^, marginal/conditional	0.148/0.710		0.149/0.710		0.110/0.701		0.177/0.727	

CE, coefficient estimate; 95% CI, 95% confidence interval; FM, fat mass; PAI, physical activity index; TOC, α-tocopherol equivalents; I (a:b) denotes the interaction effect for a and b. ^a^ Linear mixed-effects models including log plasma α-tocopherol concentrations as dependent variable, random effects of age and subject and centered metric independent variables. Model 2 considered as fixed effects: linear and quadratic age, sex, FM, smoking behavior, PAI, linear and quadratic dietary intake of α-tocopherol equivalents, use of supplements, use of lipid-modifying drugs, plasma vitamin C and disease diagnosis. Model 3 is based on model 2 and included interaction effects as additional fixed effects. Model 4 is based on model 2 but excluded records, in which the use of vitamin E and/or multivitamin supplements was reported. Model 5 is based on model 2 but only subjects with complete data records on at least seven visits were considered. Data are presented as coefficient estimates and 95% confidence intervals; ^b^ *p* values after adjusting for multiple testing: * *p* < 0.05; ** *p* < 0.01; *** *p* < 0.001; ^c^ This variable comprised the use of vitamin E and/or multivitamin supplements; ^d^ This variable combined the information on reported diagnoses of gall bladder/pancreas/chronic liver/inflammatory bowel disease and dyslipidemia.

**Table 4:** Results of linear mixed-effects models for determinants of the log α-tocopherol/total cholesterol ratio ^a,b^.

	Model 2 (*n* = 399)		Model 3 (*n* = 399)		Model 4 (*n* = 289)		Model 5 (*n* = 226)	
	CE	[95% CI]	CE	[95% CI]	CE	[95% CI]	CE	[95% CI]
Intercept	1.8 × 10^+0^ ***	[1.8 × 10^+0^, 1.8 × 10^+0^]	1.8 × 10^+0^ ***	[1.8 × 10^+0^, 1.8 × 10^+0^]	1.8 × 10^+0^ ***	[1.8 × 10^+0^, 1.8 × 10^+0^]	1.8 × 10^+0^ ***	[1.8 × 10^+0^, 1.9 × 10^+0^]
Age (years)	9.9 × 10^−3^ ***	[8.4 × 10^−3^, 1.1 × 10^−2^]	1.0 × 10^−2^ ***	[8.3 × 10^−3^, 1.2 × 10^−2^]	9.9 × 10^−3^ ***	[8.3 × 10^−3^, 1.1 × 10^−2^]	1.1 × 10^−2^ ***	[9.5 × 10^−3^, 1.3 × 10^−2^]
Age^2^ (years)	−1.2 × 10^−4^	[−2.7 × 10^−4^, 3.8 × 10^−5^]	−1.1 × 10^−4^	[−2.6 × 10^−4^, 5.3 × 10^−5^]	−1.4 × 10^−4^	[−3.0 × 10^−4^, 2.3 × 10^−5^]	−1.6 × 10^−4^	[−3.2 × 10^−4^, 9.7 × 10^−6^]
Male sex	−4.3 × 10^−3^	[−4.9 × 10^−2^, 4.1 × 10^−2^]	−3.1 × 10^−3^	[−4.8 × 10^−2^, 4.2 × 10^−2^]	3.1 × 10^−3^	[−4.1 × 10^−2^, 4.8 × 10^−2^]	−2.2 × 10^−2^	[−7.6 × 10^−2^, 3.3 × 10^−2^]
FM (kg)	2.1 × 10^−3^	[3.2 × 10^−4^, 3.8 × 10^−3^]	2.5 × 10^−3^	[4.8 × 10^−4^, 4.6 × 10^−3^]	9.3 × 10^−4^	[−9.1 × 10^−4^, 2.8 × 10^−3^]	1.1 × 10^−3^	[−1.1 × 10^−3^, 3.2 × 10^−3^]
Current/past smoking	6.8 × 10^−3^	[−3.3 × 10^−2^, 4.7 × 10^−2^]	8.0 × 10^−3^	[−3.2 × 10^−2^, 4.8 × 10^−2^]	−6.9 × 10^−3^	[−4.7 × 10^−2^, 3.3 × 10^−2^]	1.0 × 10^−2^	[−3.8 × 10^−2^, 5.9 × 10^−2^]
PAI	1.9 × 10^−2^	[−3.9 × 10^−2^, 7.7 × 10^−2^]	1.9 × 10^−2^	[−3.9 × 10^−2^, 7.8 × 10^−2^]	4.9 × 10^−3^	[−5.7 × 10^−2^, 6.7 × 10^−2^]	6.9 × 10^−2^	[2.5 × 10^−3^, 1.4 × 10^−1^]
TOC intake (mg/d)	2.1 × 10^−3^	[1.1 × 10^−4^, 4.2 × 10^−3^]	3.3 × 10^−3^	[8.5 × 10^−4^, 5.8 × 10^−3^]	2.0 × 10^−4^	[−1.9 × 10^−3^, 2.4 × 10^−3^]	2.3 × 10^−3^	[−2.4 × 10^−5^, 4.6 × 10^−3^]
TOC intake^2^ (mg/d)	−3.7 × 10^−5^	[−1.8 × 10^−4^, 1.1 × 10^−4^]	−2.5 × 10^−5^	[−1.8 × 10^−4^, 1.3 × 10^−4^]	8.8 × 10^−6^	[−1.4 × 10^−4^, 1.6 × 10^−4^]	−7.7 × 10^−5^	[−2.5 × 10^−4^, 9.6 × 10^−5^]
Use of supplements ^c^	1.2 × 10^−1^ ***	[1.0 × 10^−1^, 1.4 × 10^−1^]	1.2 × 10^−1^ ***	[1.0 × 10^−1^, 1.4 × 10^−1^]			1.1 × 10^−1^ ***	[8.6 × 10^−2^, 1.3 × 10^−1^]
Use of lipid-modifying drugs	7.5 × 10^−2^ ***	[5.3 × 10^−2^, 9.9 × 10^−2^]	7.6 × 10^−2^ ***	[5.3 × 10^−2^, 9.9 × 10^−2^]	9.9 × 10^−2^ ***	[7.4 × 10^−2^, 1.2 × 10^−1^]	7.8 × 10^−2^ ***	[5.2 × 10^−2^, 1.0 × 10^−1^]
Plasma vitamin C (µmol/L)	1.3 × 10^−3^ ***	[8.4 × 10^−4^, 1.8 × 10^−3^]	1.3 × 10^−3^ ***	[7.0 × 10^−4^, 1.9 × 10^−3^]	1.4 × 10^−3^ ***	[8.9 × 10^−4^, 2.0 × 10^−3^]	1.6 × 10^−3^ ***	[1.0 × 10^−3^, 2.2 × 10^−3^]
Disease diagnosis ^d^	3.1 × 10^−2^	[−6.9 × 10^−3^, 6.9 × 10^−2^]	3.0 × 10^−2^	[−7.3 × 10^−3^, 6.8 × 10^−2^]	−3.6 × 10^−3^	[−4.1 × 10^−2^, 3.4 × 10^−2^]	2.6 × 10^−2^	[−2.1 × 10^−2^, 7.3 × 10^−2^]
I (male sex:age)			−7.0 × 10^−4^	[−3.9 × 10^−3^, 2.5 × 10^−3^]				
I (male sex:FM)			−1.7 × 10^−3^	[−5.7 × 10^−3^, 2.3 × 10^−3^]				
I (male sex:TOC intake)			−1.6 × 10^−3^	[−5.3 × 10^−3^, 2.2 × 10^−3^]				
I (age:TOC intake)			−2.4 × 10^−5^	[−2.3 × 10^−4^, 1.8 × 10^−4^]				
I (smoking:TOC intake)			−1.7 × 10^−3^	[−5.2 × 10^−3^, 1.8 × 10^−3^]				
I (male sex:plasma vitamin C)			2.1 × 10^−4^	[−8.4 × 10^−4^, 1.3 × 10^−3^]				
I (age:plasma vitamin C)			−1.5 × 10^−5^	[−7.9 × 10^−5^, 4.9 × 10^−5^]				
R^2^, marginal/conditional	0.158/0.622		0.159/0.622		0.146/0.627		0.186/0.631	

CE, coefficient estimate; 95% CI, 95% confidence interval; FM, fat mass; PAI, physical activity index; TOC, α-tocopherol equivalents; I (a:b) denotes the interaction effect for a and b. ^a^ Linear mixed-effects models including the log α-tocopherol/total cholesterol ratio as dependent variable, random effects of age and subject and centered metric independent variables. Model 2 considered as fixed effects: linear and quadratic age, sex, FM, smoking behavior, PAI, linear and quadratic dietary intake of α-tocopherol equivalents, use of supplements, use of lipid-modifying drugs, plasma vitamin C and disease diagnosis. Model 3 is based on model 2 and included interaction effects as additional fixed effects. Model 4 is based on model 2 but excluded records, in which the use of vitamin E and/or multivitamin supplements was reported. Model 5 is based on model 2 but only subjects with complete data records on at least seven visits were considered. Data are presented as coefficient estimates and 95% confidence intervals; ^b^ *p* values after adjusting for multiple testing: *** *p* < 0.001; ^c^ This variable comprised the use of vitamin E and/or multivitamin supplements; ^d^ This variable combined the information on reported diagnoses of gall bladder/pancreas/chronic liver/inflammatory bowel disease and dyslipidemia.

## Supplementary Materials

The following are available online at https://www.mdpi.com/2072-6643/12/10/2944/s1, Supplementary Table S1: Results of linear mixed-effects models for determinants of vitamin C plasma concentrations after excluding model-specific outliers, Supplementary Table S2: Results of linear mixed-effects models for determinants of log plasma α-tocopherol concentrations including serum cholesterol, Supplementary Table S3: Results of linear mixed-effects models for determinants of log plasma α-tocopherol concentrations after excluding model-specific outliers, Supplementary Table S4: Results of linear mixed-effects models for determinants of the log α-tocopherol/total cholesterol ratio after excluding model-specific outliers.

## Abbreviations

95% CI	95% confidence interval
BIA	bioelectrical impedance analysis
BMI	body mass index
DRVs	dietary reference values
FFM	fat-free mass
FM	fat mass
GISELA	longitudinal study on nutrition and health status of senior citizens in Giessen
PAI	physical activity index
PRI	Population Reference Intakes
PUFA	polyunsaturated fatty acids
