# Supplementary material for: Interrelation between Plasma Concentrations of Vitamins C and E along the Trajectory of Ageing in Consideration of Lifestyle and Body Composition: A Longitudinal Study over Two Decades
Source: Nutrients. 2020 Sep 25;12(10):2944. doi: 10.3390/nu12102944 (PMC7599873; doi:10.3390/nu12102944)
Supplement: Supplementary file 1 [file nutrients-12-02944-s001.pdf]

Supplementary Table 1. Results of linear mixed-effects models for determinants of vitamin C plasma concentrations after excluding model-specific outliers [a, b].

	**Model 2 (*n* = 396, 2762 records)**		**Model 3 (*n* = 396, 2766 records)**		**Model 4 (*n* = 297, 1804 records)**		**Model 5 (*n* = 226, 1990 records)**	
	**CE**	**[95% CI]**	**CE**	**[95% CI]**	**CE**	**[95% CI]**	**CE**	**[95% CI]**
Intercept	$7.5\times10^{+1}$ ***	[$7.3\times10^{+1}$, $7.8\times10^{+1}$]	$7.5\times10^{+1}$ ***	[$7.2\times10^{+1}$, $7.7\times10^{+1}$]	$7.5\times10^{+1}$ ***	[$7.3\times10^{+1}$, $7.8\times10^{+1}$]	$7.5\times10^{+1}$ ***	[$7.2\times10^{+1}$, $7.9\times10^{+1}$]
Age (years)	-1.3×10^{-2}	[-1.2×10^{-1}, 9.4×10^{-2}]	-3.9×10^{-2}	[-1.7×10^{-1}, 9.5×10^{-2}]	-1.0×10^{-2}	[-1.4×10^{-1}, 1.2×10^{-1}]	6.0×10^{-3}	[-1.1×10^{-1}, 1.2×10^{-1}]
Age² (years)	2.2×10^{-3}	[-7.7×10^{-3}, 1.2×10^{-2}]	2.0×10^{-3}	[-7.9×10^{-3}, 1.2×10^{-2}]	-4.0×10^{-3}	[-1.6×10^{-2}, 8.4×10^{-3}]	4.2×10^{-3}	[-6.6×10^{-3}, 1.5×10^{-2}]
Male sex	-4.3×10^{-1}	[$-5.0\times10^{+0}$, $4.1\times10^{+0}$]	$-4.9\times10^{+0}$	[$-1.1\times10^{+1}$, 7.3×10^{-1}]	$-2.0\times10^{+0}$	[$-6.9\times10^{+0}$, $3.0\times10^{+0}$]	-5.5×10^{-1}	[$-6.1\times10^{+0}$, $5.0\times10^{+0}$]
FFM (kg)	-4.7×10^{-1} ***	[-6.9×10^{-1}, -2.4×10^{-1}]	-6.8×10^{-1} ***	[-9.6×10^{-1}, -4.0×10^{-1}]	-3.8×10^{-1} *	[-6.4×10^{-1}, -1.3×10^{-1}]	-4.6×10^{-1} **	[-7.2×10^{-1}, -2.0×10^{-1}]
Current/past smoking	$-3.0\times10^{+0}$	[$-5.7\times10^{+0}$, -2.0×10^{-1}]	$-3.4\times10^{+0}$	[$-6.2\times10^{+0}$, -6.0×10^{-1}]	$-2.3\times10^{+0}$	[$-5.2\times10^{+0}$, 6.3×10^{-1}]	$-1.9\times10^{+0}$	[$-5.4\times10^{+0}$, $1.5\times10^{+0}$]
PAI	$6.0\times10^{+0}$ *	[$2.4\times10^{+0}$, $9.6\times10^{+0}$]	$6.4\times10^{+0}$ **	[$2.8\times10^{+0}$, $1.0\times10^{+1}$]	$9.9\times10^{+0}$ ***	[$5.4\times10^{+0}$, $1.4\times10^{+1}$]	$5.7\times10^{+0}$ ‡	[$1.5\times10^{+0}$, $1.0\times10^{+1}$]
Vitamin C intake (mg/d)	1.4×10^{-1}	[1.7×10^{-2}, 2.6×10^{-1}]	1.1×10^{-1}	[-3.9×10^{-2}, 2.6×10^{-1}]	1.5×10^{-1}	[-1.9×10^{-3}, 3.0×10^{-1}]	1.1×10^{-1}	[-3.4×10^{-2}, 2.6×10^{-1}]
Vitamin C intake² (mg/d)	-7.1×10^{-3}	[-1.4×10^{-2}, -4.0×10^{-4}]	-8.0×10^{-3}	[-1.5×10^{-2}, -1.3×10^{-3}]	-8.6×10^{-3}	[-1.7×10^{-2}, -3.2×10^{-4}]	-5.1×10^{-3}	[-1.3×10^{-2}, 3.4×10^{-3}]
Use of supplements [c]	$3.7\times10^{+0}$ ***	[$2.6\times10^{+0}$, $4.7\times10^{+0}$]	$3.7\times10^{+0}$ ***	[$2.6\times10^{+0}$, $4.7\times10^{+0}$]			$3.9\times10^{+0}$ ***	[$2.7\times10^{+0}$, $5.1\times10^{+0}$]
Use of lipid-modifying drugs	3.1×10^{-1}	[$-1.2\times10^{+0}$, $1.8\times10^{+0}$]	2.3×10^{-1}	[$-1.3\times10^{+0}$, $1.7\times10^{+0}$]	-6.1×10^{-1}	[$-2.4\times10^{+0}$, $1.2\times10^{+0}$]	5.6×10^{-1}	[$-1.1\times10^{+0}$, $2.2\times10^{+0}$]
Plasma TOC (µmol/L)	1.5×10^{-1} ***	[9.7×10^{-2}, 1.9×10^{-1}]	1.4×10^{-1} ***	[8.8×10^{-2}, 2.0×10^{-1}]	2.1×10^{-1} ***	[1.4×10^{-1}, 2.8×10^{-1}]	2.0×10^{-1} ***	[1.4×10^{-1}, 2.7×10^{-1}]
Disease diagnosis [d]	$-1.0\times10^{+0}$	[$-3.7\times10^{+0}$, $1.6\times10^{+0}$]	-8.1×10^{-1}	[$-3.4\times10^{+0}$, $1.8\times10^{+0}$]	$-2.2\times10^{+0}$	[-4.9×10+0, 6.1×10^{-1}]	$-1.5\times10^{+0}$	[$-4.8\times10^{+0}$, $1.9\times10^{+0}$]
I (male sex : age)			-1.8×10^{-2}	[-2.4×10^{-1}, 2.1×10^{-1}]				
I (male sex : FFM)			6.0×10^{-1}	[1.5×10^{-1}, $1.1\times10^{+0}$]				
I (male sex : VC intake)			-7.6×10^{-2}	[-2.7×10^{-1}, 1.2×10^{-1}]				
I (age : VC intake)			1.1×10^{-2}	[-5.1×10^{-4}, 2.2×10^{-2}]				
I (smoking : VC intake)			1.5×10^{-1}	[-3.0×10^{-2}, 3.3×10^{-1}]				
I (male sex : plasma TOC)			4.7×10^{-2}	[-6.6×10^{-2}, 1.6×10^{-1}]				
I (age : plasma TOC)			1.1×10^{-3}	[-5.7×10^{-3}, 8.0×10^{-3}]				
R^2, marginal / conditional	0.125 / 0.686		0.134 / 0.685		0.131 / 0.658		0.139 / 0.681	

CE, coefficient estimate; 95% CI, 95% confidence interval; FFM, fat-free mass; PAI, physical activity index; VC, vitamin C; I (a : b) denotes the interaction effect for a and b. [a] Results are presented as coefficient estimates and 95% confidence intervals; [b] *P* values after adjusting for multiple testing: ‡ $p < 0.10$; * $p < 0.05$; ** $p < 0.01$; *** $p < 0.001$; [c] This parameter comprised the use of vitamin C and/or multivitamin supplements; [d] This variable combined the information on the presence of gall bladder/pancreas/chronic liver/inflammatory bowel disease and dyslipidemia.

Supplementary Table 2. Results of linear mixed-effects models for determinants of log plasma α-tocopherol concentrations including serum cholesterol [a, b].

	Model 2 (n = 399)		Model 3 (n = 399)		Model 4 (n = 289)		Model 5 (n = 226)	
	**CE**	**[95% CI]**	**CE**	**[95% CI]**	**CE**	**[95% CI]**	**CE**	**[95% CI]**
Intercept	$3.5\times10^{+0}$ ***	[$3.5\times10^{+0}$, $3.6\times10^{+0}$]	$3.5\times10^{+0}$ ***	[$3.5\times10^{+0}$, $3.5\times10^{+0}$]	$3.5\times10^{+0}$ ***	[$3.5\times10^{+0}$, $3.5\times10^{+0}$]	$3.5\times10^{+0}$ ***	[$3.5\times10^{+0}$, $3.6\times10^{+0}$]
Age (years)	7.9×10^{-3} ***	[6.3×10^{-3}, 9.4×10^{-3}]	8.6×10^{-3} ***	[6.7×10^{-3}, 1.0×10^{-2}]	8.0×10^{-3} ***	[6.4×10^{-3}, 9.5×10^{-3}]	9.1×10^{-3} ***	[7.4×10^{-3}, 1.1×10^{-2}]
Age² (years)	-1.5×10^{-4}	[-3.0×10^{-4}, -3.1×10^{-6}]	-1.3×10^{-4}	[-2.8×10^{-4}, 2.1×10^{-5}]	-1.8×10^{-4}	[-3.3×10^{-4}, -2.4×10^{-5}]	-1.8×10^{-4}	[-3.4×10^{-4}, -1.9×10^{-5}]
Male sex	-5.5×10^{-2}	[-1.0×10^{-1}, -9.3×10^{-3}]	-5.2×10^{-2}	[-9.9×10^{-2}, -5.6×10^{-3}]	-5.0×10^{-2}	[-9.5×10^{-2}, -5.2×10^{-3}]	-8.4×10^{-2} *	[-1.4×10^{-1}, -2.9×10^{-2}]
FM (kg)	1.3×10^{-3}	[-4.6×10^{-4}, 3.1×10^{-3}]	1.7×10^{-3}	[-3.1×10^{-4}, 3.8×10^{-3}]	3.5×10^{-4}	[-1.5×10^{-3}, 2.2×10^{-3}]	2.0×10^{-4}	[-1.9×10^{-3}, 2.3×10^{-3}]
Current/past smoking	2.2×10^{-2}	[-1.9×10^{-2}, 6.3×10^{-2}]	2.4×10^{-2}	[-1.7×10^{-2}, 6.4×10^{-2}]	1.2×10^{-2}	[-2.8×10^{-2}, 5.2×10^{-2}]	2.7×10^{-2}	[-2.2×10^{-2}, 7.7×10^{-2}]
PAI	2.7×10^{-2}	[-2.9×10^{-2}, 8.3×10^{-2}]	2.8×10^{-2}	[-2.8×10^{-2}, 8.4×10^{-2}]	1.4×10^{-2}	[-4.3×10^{-2}, 7.3×10^{-2}]	8.4×10^{-2}	[2.0×10^{-2}, 1.5×10^{-1}]
TOC intake (mg/d)	2.1×10^{-3}	[1.6×10^{-4}, 4.0×10^{-3}]	3.2×10^{-3}	[8.8×10^{-4}, 5.6×10^{-3}]	3.3×10^{-4}	[-1.7×10^{-3}, 2.4×10^{-3}]	2.5×10^{-3}	[2.6×10^{-4}, 4.7×10^{-3}]
TOC intake² (mg/d)	-5.8×10^{-5}	[-2.0×10^{-4}, 8.3×10^{-5}]	-3.7×10^{-5}	[-1.8×10^{-4}, 1.1×10^{-4}]	-5.4×10^{-6}	[-1.4×10^{-4}, 1.3×10^{-4}]	-1.2×10^{-4}	[-2.8×10^{-4}, 4.9×10^{-5}]
Use of supplements [c]	1.2×10^{-1} ***	[1.0×10^{-1}, 1.4×10^{-1}]	1.2×10^{-1} ***	[1.0×10^{-1}, 1.4×10^{-1}]			1.1×10^{-1} ***	[8.7×10^{-2}, 1.3×10^{-1}]
Use of lipid-modifying drugs	1.7×10^{-2}	[-6.2×10^{-3}, 4.0×10^{-2}]	1.7×10^{-2}	[-5.7×10^{-3}, 4.1×10^{-2}]	3.8×10^{-2} *	[1.4×10^{-2}, 6.3×10^{-2}]	1.5×10^{-2}	[-1.0×10^{-2}, 4.1×10^{-2}]
Plasma vitamin C (µmol/L)	1.4×10^{-3} ***	[9.7×10^{-4}, 1.9×10^{-3}]	1.4×10^{-3} ***	[8.3×10^{-4}, 2.0×10^{-3}]	1.5×10^{-3} ***	[1.0×10^{-3}, 2.0×10^{-3}]	1.7×10^{-3} ***	[1.2×10^{-3}, 2.3×10^{-3}]
Disease diagnosis [d]	6.5×10^{-2} *	[2.6×10^{-2}, 1.0×10^{-1}]	6.5×10^{-2} *	[2.6×10^{-2}, 1.0×10^{-1}]	2.9×10^{-2}	[-8.1×10^{-3}, 6.6×10^{-2}]	5.4×10^{-2}	[6.0×10^{-3}, 1.0×10^{-1}]
Serum cholesterol (mmol/L)	1.0×10^{-1} ***	[9.3×10^{-2}, 1.1×10^{-1}]	1.0×10^{-1} ***	[9.3×10^{-2}, 1.1×10^{-1}]	1.0×10^{-1} ***	[9.3×10^{-2}, 1.1×10^{-1}]	9.9×10^{-2} ***	[8.9×10^{-2}, 1.1×10^{-1}]
I (male sex : age)			-2.0×10^{-3}	[-5.2×10^{-3}, 1.2×10^{-3}]				
I (male sex : FM)			-1.5×10^{-3}	[-5.6×10^{-3}, 2.4×10^{-3}]				
I (male sex : TOC intake)			-1.4×10^{-3}	[-5.0×10^{-3}, 2.2×10^{-3}]				
I (age : TOC intake)			-7.3×10^{-5}	[-2.7×10^{-4}, 1.2×10^{-4}]				
I (smoking : TOC intake)			-1.7×10^{-3}	[-5.1×10^{-3}, 1.6×10^{-3}]				
I (male sex : plasma vitamin C)			2.6×10^{-4}	[-7.5×10^{-4}, 1.3×10^{-3}]				
I (age : plasma vitamin C)			-2.5×10^{-5}	[-8.6×10^{-5}, 3.7×10^{-5}]				
R^2, marginal / conditional	0.302 / 0.719		0.302 / 0.718		0.312 / 0.724		0.342 / 0.734	

CE, coefficient estimate; 95% CI, 95% confidence interval; FM, fat mass; PAI, physical activity index; TOC, α-tocopherol equivalents; I (a : b) denotes the interaction effect for a and b. [a] Results are presented as coefficient estimates and 95% confidence intervals; [b] P values after adjusting for multiple testing: * $p < 0.05$; *** $p < 0.001$; [c] This parameter comprised the use of vitamin E and/or multivitamin supplements; [d] This variable combined the information on the presence of gall bladder/pancreas/chronic liver/inflammatory bowel disease and dyslipidemia.

Supplementary Table 3. Results of linear mixed-effects models for determinants of log plasma α-tocopherol concentrations after excluding model-specific outliers [a, b].

	**Model 2 (*n* = 392, 2743 records)**		**Model 3 (*n* = 392, 2746 records)**		**Model 4 (*n* = 283, 1749 records)**		**Model 5 (*n* = 226, 1986 records)**	
	**CE**	**[95% CI]**	**CE**	**[95% CI]**	**CE**	**[95% CI]**	**CE**	**[95% CI]**
Intercept	$3.5\times10^{+0}$ ***	[$3.5\times10^{+0}$, $3.6\times10^{+0}$]	$3.5\times10^{+0}$ ***	[$3.5\times10^{+0}$, $3.6\times10^{+0}$]	$3.5\times10^{+0}$ ***	[$3.5\times10^{+0}$, $3.5\times10^{+0}$]	$3.5\times10^{+0}$ ***	[$3.5\times10^{+0}$, $3.6\times10^{+0}$]
Age (years)	4.4×10^{-3} ***	[2.7×10^{-3}, 6.0×10^{-3}]	5.5×10^{-3} ***	[3.6×10^{-3}, 7.5×10^{-3}]	5.1×10^{-3} ***	[3.5×10^{-3}, 6.7×10^{-3}]	5.7×10^{-3} ***	[3.9×10^{-3}, 7.4×10^{-3}]
Age^2 (years)	-1.4×10^{-4}	[-2.9×10^{-4}, 4.2×10^{-7}]	-1.2×10^{-4}	[-2.6×10^{-4}, 3.1×10^{-5}]	-1.6×10^{-4}	[-3.2×10^{-4}, -1.2×10^{-5}]	-1.3×10^{-4}	[-2.8×10^{-4}, 2.1×10^{-5}]
Male sex	-1.1×10^{-1} ***	[-1.7×10^{-1}, -6.1×10^{-2}]	-1.1×10^{-1} **	[-1.6×10^{-1}, -5.3×10^{-2}]	-1.1×10^{-1} ***	[-1.6×10^{-1}, -5.8×10^{-2}]	-1.5×10^{-1} ***	[-2.2×10^{-1}, -8.3×10^{-2}]
FM (kg)	2.9×10^{-4}	[-1.6×10^{-3}, 2.1×10^{-3}]	4.7×10^{-4}	[-1.7×10^{-3}, 2.6×10^{-3}]	4.1×10^{-4}	[-1.6×10^{-3}, 2.4×10^{-3}]	2.7×10^{-4}	[-2.0×10^{-3}, 2.5×10^{-3}]
Current/past smoking	3.9×10^{-2}	[-7.9×10^{-3}, 8.7×10^{-2}]	4.1×10^{-2}	[-6.4×10^{-3}, 8.8×10^{-2}]	3.0×10^{-2}	[-1.8×10^{-2}, 7.7×10^{-2}]	5.0×10^{-2}	[-9.2×10^{-3}, 1.1×10^{-1}]
PAI	1.9×10^{-2}	[-3.2×10^{-2}, 6.9×10^{-2}]	1.9×10^{-2}	[-3.2×10^{-2}, 6.9×10^{-2}]	4.5×10^{-3}	[-5.0×10^{-2}, 5.9×10^{-2}]	7.6×10^{-2}	[1.8×10^{-2}, 1.3×10^{-1}]
TOC intake (mg/d)	7.3×10^{-4}	[-1.0×10^{-3}, 2.5×10^{-3}]	1.1×10^{-3}	[-1.1×10^{-3}, 3.2×10^{-3}]	3.6×10^{-5}	[-1.8×10^{-3}, 1.9×10^{-3}]	7.0×10^{-4}	[-1.3×10^{-3}, 2.7×10^{-3}]
TOC $intake^2$ (mg/d)	-2.7×10^{-5}	[-1.5×10^{-4}, 9.8×10^{-5}]	3.4×10^{-7}	[-1.3×10^{-4}, 1.3×10^{-4}]	3.0×10^{-5}	[-9.9×10^{-5}, 1.6×10^{-4}]	-2.6×10^{-5}	[-1.8×10^{-4}, 1.2×10^{-4}]
Use of supplements [c]	9.6×10^{-2} ***	[8.0×10^{-2}, 1.1×10^{-1}]	9.6×10^{-2} ***	[8.0×10^{-2}, 1.1×10^{-1}]			9.3×10^{-2} ***	[7.5×10^{-2}, 1.1×10^{-1}]
Use of lipid-modifying drugs	-5.6×10^{-2} ***	[-7.7×10^{-2}, -3.5×10^{-2}]	-5.6×10^{-2} ***	[-7.6×10^{-2}, -3.5×10^{-2}]	-4.2×10^{-2} **	[-6.4×10^{-2}, -1.8×10^{-2}]	-6.6×10^{-2} ***	[-8.9×10^{-2}, -4.3×10^{-2}]
Plasma vitamin C (µmol/L)	1.6×10^{-3} ***	[1.2×10^{-3}, 2.1×10^{-3}]	1.5×10^{-3} ***	[9.9×10^{-4}, 2.0×10^{-3}]	1.4×10^{-3} ***	[9.5×10^{-4}, 1.9×10^{-3}]	1.8×10^{-3} ***	[1.3×10^{-3}, 2.3×10^{-3}]
Disease diagnosis [d]	1.1×10^{-1} ***	[6.7×10^{-2}, 1.6×10^{-1}]	1.1×10^{-1} ***	[6.8×10^{-2}, 1.6×10^{-1}]	8.1×10^{-2} **	[3.6×10^{-2}, 1.2×10^{-1}]	8.9×10^{-2} *	[3.1×10^{-2}, 1.5×10^{-1}]
I (male sex : age)			-3.0×10^{-3}	[-6.4×10^{-3}, 3.7×10^{-4}]				
I (male sex : FM)			-1.9×10^{-4}	[-4.4×10^{-3}, 3.9×10^{-3}]				
I (male sex : TOC intake)			2.9×10^{-4}	[-2.9×10^{-3}, 3.5×10^{-3}]				
I (age : TOC intake)			-1.3×10^{-4}	[-3.1×10^{-4}, 5.4×10^{-5}]				
I (smoking : TOC intake)			-1.3×10^{-3}	[-4.4×10^{-3}, 1.7×10^{-3}]				
I (male sex : plasma vitamin C)			7.2×10^{-4}	[-2.2×10^{-4}, 1.6×10^{-3}]				
I (age : plasma vitamin C)			-8.1×10^{-5}	[-1.4×10^{-4}, -2.1×10^{-5}]				
R^2, marginal / conditional	0.148 / 0.784		0.150 / 0.783		0.127 / 0.777		0.179 / 0.798	

CE, coefficient estimate; 95% CI, 95% confidence interval; FM, fat mass; PAI, physical activity index; TOC, α-tocopherol equivalents; I (a : b) denotes the interaction effect for a and b. [a] Data are presented as coefficient estimates and 95% confidence intervals; [b] *P* values after adjusting for multiple testing: * $p < 0.05$; ** $p < 0.01$; *** $p < 0.001$; [c] This parameter comprised the use of vitamin E and/or multivitamin supplements; [d] This variable combined the information on reported diagnoses of gall bladder/pancreas/chronic liver/inflammatory bowel disease and dyslipidemia.

Supplementary Table 4. Results of linear mixed-effects models for determinants of the log α-tocopherol/total cholesterol ratio after excluding model-specific outliers [a, b].

	**Model 2 (n = 393, 2748 records)**		**Model 3 (n = 394, 2751 records)**		**Model 4 (n = 285, 1760 records)**		**Model 5 (n = 226, 1982 records)**	
	**CE**	**[95% CI]**	**CE**	**95% CI**	**CE**	**[95% CI]**	**CE**	**[95% CI]**
Intercept	$1.8\times10^{+0}$ ***	[$1.8\times10^{+0}$, $1.8\times10^{+0}$]	$1.8\times10^{+0}$ ***	[$1.8\times10^{+0}$, $1.8\times10^{+0}$]	$1.8\times10^{+0}$ ***	[$1.8\times10^{+0}$, $1.8\times10^{+0}$]	$1.8\times10^{+0}$ ***	[$1.8\times10^{+0}$, $1.9\times10^{+0}$]
Age (years)	9.5×10^{-3} ***	[8.1×10^{-3}, 1.1×10^{-2}]	9.9×10^{-3} ***	[8.2×10^{-3}, 1.2×10^{-2}]	9.7×10^{-3} ***	[8.3×10^{-3}, 1.1×10^{-2}]	1.1×10^{-2} ***	[9.5×10^{-3}, 1.2×10^{-2}]
Age^2 (years)	-1.7×10^{-4} ‡	[-3.1×10^{-4}, -4.0×10^{-5}]	-1.6×10^{-4}	[-3.0×10^{-4}, -2.5×10^{-5}]	-1.7×10^{-4}	[-3.1×10^{-4}, -3.4×10^{-5}]	-1.6×10^{-4}	[-3.0×10^{-4}, -2.0×10^{-5}]
Male sex	-8.0×10^{-3}	[-5.1×10^{-2}, 3.5×10^{-2}]	-5.4×10^{-3}	[-4.9×10^{-2}, 3.8×10^{-2}]	1.6×10^{-5}	[-4.3×10^{-2}, 4.3×10^{-2}]	-2.7×10^{-2}	[-8.0×10^{-2}, 2.6×10^{-2}]
FM (kg)	1.2×10^{-3}	[-4.2×10^{-4}, 2.8×10^{-3}]	1.7×10^{-3}	[-1.6×10^{-4}, 3.6×10^{-3}]	6.6×10^{-4}	[-1.0×10^{-3}, 2.4×10^{-3}]	6.1×10^{-4}	[-1.3×10^{-3}, 2.6×10^{-3}]
Current/past smoking	3.8×10^{-3}	[-3.5×10^{-2}, 4.2×10^{-2}]	5.5×10^{-3}	[-3.3×10^{-2}, 4.4×10^{-2}]	-3.8×10^{-3}	[-4.3×10^{-2}, 3.5×10^{-2}]	1.3×10^{-2}	[-3.5×10^{-2}, 6.0×10^{-2}]
PAI	-2.5×10^{-3}	[-5.1×10^{-2}, 4.6×10^{-2}]	-2.4×10^{-3}	[-5.1×10^{-2}, 4.6×10^{-2}]	-6.8×10^{-3}	[-6.0×10^{-2}, 4.6×10^{-2}]	5.2×10^{-2}	[-3.6×10^{-3}, 1.1×10^{-1}]
TOC intake (mg/d)	1.2×10^{-3}	[-5.2×10^{-4}, 2.9×10^{-3}]	2.1×10^{-3}	[5.3×10^{-5}, 4.2×10^{-3}]	-1.4×10^{-4}	[-2.0×10^{-3}, 1.7×10^{-3}]	1.8×10^{-3}	[-1.8×10^{-4}, 3.7×10^{-3}]
TOC intake^2 (mg/d)	4.2×10^{-6}	[-1.2×10^{-4}, 1.3×10^{-4}]	1.2×10^{-5}	[-1.1×10^{-4}, 1.4×10^{-4}]	5.1×10^{-5}	[-7.4×10^{-5}, 1.7×10^{-4}]	-3.8×10^{-5}	[-1.8×10^{-4}, 1.1×10^{-4}]
Use of supplements [c]	1.1×10^{-1} ***	[9.4×10^{-2}, 1.2×10^{-1}]	1.1×10^{-1} ***	[9.5×10^{-2}, 1.3×10^{-1}]			1.0×10^{-1} ***	[8.6×10^{-2}, 1.2×10^{-1}]
Use of lipid-modifying drugs	7.9×10^{-2} ***	[5.9×10^{-2}, 9.9×10^{-2}]	7.9×10^{-2} ***	[6.0×10^{-2}, 9.9×10^{-2}]	1.0×10^{-1} ***	[7.9×10^{-2}, 1.2×10^{-1}]	7.3×10^{-2} ***	[5.1×10^{-2}, 9.5×10^{-2}]
Plasma vitamin C (μmol/L)	1.1×10^{-3} ***	[6.4×10^{-4}, 1.5×10^{-3}]	1.1×10^{-3} ***	[5.7×10^{-4}, 1.6×10^{-3}]	1.3×10^{-3} ***	[8.6×10^{-4}, 1.8×10^{-3}]	1.2×10^{-3} ***	[6.9×10^{-4}, 1.6×10^{-3}]
Disease diagnosis [d]	2.2×10^{-2}	[-1.4×10^{-2}, 5.9×10^{-2}]	2.2×10^{-2}	[-1.4×10^{-2}, 5.8×10^{-2}]	-2.7×10^{-3}	[-3.9×10^{-2}, 3.4×10^{-2}]	2.1×10^{-2}	[-2.5×10^{-2}, 6.7×10^{-2}]
I (male sex : age)			-1.1×10^{-3}	[-3.9×10^{-3}, 1.8×10^{-3}]				
I (male sex : FM)			-1.7×10^{-3}	[-5.3×10^{-3}, 1.9×10^{-3}]				
I (male sex : TOC intake)			1.4×10^{-4}	[-3.0×10^{-3}, 3.3×10^{-3}]				
I (age : TOC intake)			7.2×10^{-6}	[-1.7×10^{-4}, 1.8×10^{-4}]				
I (smoking : TOC intake)			-2.4×10^{-3}	[-5.3×10^{-3}, 5.5×10^{-4}]				
I (male sex : plasma vitamin C)			1.7×10^{-4}	[-7.2×10^{-4}, 1.1×10^{-3}]				
I (age : plasma vitamin C)			-4.6×10^{-5}	[-1.0×10^{-4}, 9.0×10^{-6}]				
R^2, marginal / conditional	0.159 / 0.709		0.160 / 0.709		0.165 / 0.704		0.190 / 0.715	

CE, coefficient estimate; 95% CI, 95% confidence interval; FM, fat mass; PAI, physical activity index; TOC, α-tocopherol equivalents; I (a : b) denotes the interaction effect for a and b. [a] Results are presented as coefficient estimates and 95% confidence intervals; [b] P values after adjusting for multiple testing: ‡ $p < 0.10$; *** $p < 0.001$; [c] This parameter comprised the use of vitamin E and/or multivitamin supplements; [d] This variable combined the information on the presence of gall bladder/pancreas/chronic liver/inflammatory bowel disease and dyslipidemia.
